# Factors Associated With the Discussion of Fertility Preservation in a Cohort of 1,357 Young Breast Cancer Patients Receiving Chemotherapy

**DOI:** 10.3389/fonc.2021.701620

**Published:** 2021-09-28

**Authors:** Alice Hours, Aullene Toussaint, Victoire De Castelbajac, Camille Sautter, Julie Borghese, Sophie Frank, Florence Coussy, Enora Laas, Beatriz Grandal, Elise Dumas, Eric Daoud, Julien Guerin, Thomas Balezeau, Jean-Guillaume Feron, Virginie Fourchotte, Youlia Kirova, Florence Lerebours, Jean-Yves Pierga, Eugénie Guillot, Pietro Santulli, Michael Grynberg, Charlotte Sonigo, Emmanuel Reyrat, Pauline Soibinet-Oudot, Fabien Reyal, Anne-Sophie Hamy

**Affiliations:** ^1^ Department of Surgery, Institut Curie, University Paris, Paris, France; ^2^ Department of Medical Oncology, Institut Curie, University Paris, Paris, France; ^3^ Residual Tumor & Response to Treatment Laboratory, RT2Lab, Translational Research Department, INSERM, U932 Immunity and Cancer, Paris, France; ^4^ Sénopole Hôpital Saint Louis, Assistance Publique - Hôpitaux de Paris, University Paris, Paris, France; ^5^ Data Factory, Institut Curie, Paris, France; ^6^ Department of Radiation Therapy, Institut Curie, Paris, France; ^7^ Department of Obstetrics and Gynecology, Hôpital Cochin, University Paris, Paris, France; ^8^ Department of Reproductive Medicine and Fertility Preservation, Hôpital Antoine Béclère, Hôpitaux Universitaires Paris Sud, Assistance Publique - Hôpitaux de Paris, Clamart, France; ^9^ Department of Reproductive Medicine and Fertility Preservation, Hôpital Jean Verdier, Bondy, France; ^10^ Department of Data and Informatics, Unicancer, Paris, France; ^11^ Department of Medical Oncology, Godinot Institute, Reims, France

**Keywords:** breast cancer, fertility preservation, discussion, chemotherapy, oncofertility

## Abstract

**Purpose:**

Female breast cancer (BC) patients exposed to gonadotoxic chemotherapy are at risk of future infertility. There is evidence of disparities in the discussion of fertility preservation for these patients. The aim of the study was to identify factors influencing the discussion of fertility preservation (FP).

**Material and Methods:**

We analyzed consecutive BC patients treated by chemotherapy at Institut Curie from 2011-2017 and aged 18-43 years at BC diagnosis. The discussion of FP was classified in a binary manner (discussion/no discussion), based on mentions present in the patient’s electronic health record (EHR) before the initiation of chemotherapy. The associations between FP discussion and the characteristics of patients/tumors and healthcare practitioners were investigated by logistic regression analysis.

**Results:**

The median age of the 1357 patients included in the cohort was 38.7 years, and median tumor size was 30.3 mm. The distribution of BC subtypes was as follows: 702 luminal BCs (58%), 241 triple-negative breast cancers (TNBCs) (20%), 193 HER2^+^/HR^+^ (16%) and 81 HER2^+^/HR^-^ (6%). All patients received chemotherapy in a neoadjuvant (*n*=611, 45%) or adjuvant (*n*= 744, 55%) setting. A discussion of FP was mentioned for 447 patients (33%). Earlier age at diagnosis (discussion: 34.4 years *versus* no discussion: 40.5 years), nulliparity (discussion: 62% *versus* no discussion: 38%), and year of BC diagnosis were the patient characteristics significantly associated with the mention of FP discussion. Surgeons and female physicians were the most likely to mention FP during the consultation before the initiation of chemotherapy (discussion: 22% and 21%, respectively). The likelihood of FP discussion increased significantly over time, from 15% in 2011 to 45% in 2017. After multivariate analysis, FP discussion was significantly associated with younger age, number of children before BC diagnosis, physicians’ gender and physicians’ specialty.

**Conclusion:**

FP discussion rates are low and are influenced by patient and physician characteristics. There is therefore room for improvement in the promotion and systematization of FP discussion.

## 1 Introduction

Breast cancer (BC) is the most frequent cancer in women (1), and about 7% of BC diagnoses concern women under the age of 40 years (2). Survival rates are continually improving, thanks to advances in early detection and treatment. Mean age at first pregnancy is continuing to increase, due to changes in society, and the question of fertility and pregnancy after BC is therefore being raised increasingly frequently (3).

Oncological treatments may impair the fertility of premenopausal patients with BC. Chemotherapy may induce premature ovarian failure, depending on the woman’s age and the drugs used, their dose and the duration of treatment (4). Adjuvant endocrine therapy, which is generally recommended for five years in patients with hormone-responsive cancers, can also delay parenthood, due to the potential teratogenicity of the treatment (5).

A number of fertility preservation (FP) techniques are available, and the freezing of embryos or oocytes after controlled stimulation for future *in vitro* fertilization procedures is the most frequently used (6). If this is unfeasible or if ovarian stimulation is contraindicated, ovarian tissue cryopreservation of oocyte/embryo vitrification after the *in vitro* maturation of oocytes recovered from small antral follicles may be used as an alternative (7).

Previous studies have suggested that many BC patients are interested in maintaining their future fertility at the time of diagnosis. However, they do not systematically receive information about the fertility risks of treatment and fertility preservation options (8), with such discussion occurring in 30 to 70% of patients (9, 10). The American Society of Clinical Oncology recommends that physicians question newly diagnosed cancer patients as soon as possible about their desire for future fertility, and that interested patients be immediately referred to specialists in fertility preservation techniques, when appropriate (6). In France, the National Cancer Plan 2014-2019 highlighted the need for systematic and appropriate information on fertility preservation and promoted the concept of oncofertility (11).

Publications to date on the factors predictive of FP discussion in BC are mostly limited to small qualitative studies. Disparities in referral patterns and access to FP have been observed with respect to the demographic, clinical and socioeconomic characteristics of patients. A few studies have shown that patient age, and parity, the type of treatment, type of center and physician characteristics may affect the likelihood of FP discussion (9, 10, 12).

The objective of this study was to identify the factors associated with FP discussion in a population of women receiving chemotherapy for BC to improve patient counselling and timely access to FP services.

## 2 Materials and Methods

### 2.1 Study Design

We analyzed a cohort of female patients with invasive BC aged between 18 and 43 years at the time of BC diagnosis, treated by chemotherapy at Institut Curie between January 1, 2011 and September 30, 2017. The upper limit of 43 years was chosen as this is the maximum age for reimbursement of assisted reproductive technology in France. In the study, we also used the 37 years as a cut-off point, as it has been shown that the age of 37 years is correlated with an accelerated disappearance of ovarian follicles in mid-life (13, 14). The study was conducted at two centers: the Institut Curie centers at Paris and Saint Cloud.

The cohort was constructed with the ConSore (15) search engine, a next-generation data analysis program developed by UNICANCER and allowing both requests with structured criteria and natural language processing for semantic searches (flow chart in **Supplementary Table 1**).

The exclusion criteria were another cancer before BC, distant metastases at diagnosis or within six months of diagnosis, bilateral breast cancer, refusal of treatment, hysterectomy, tubal sterilization or bilateral ovariectomy performed before diagnosis, patient refusal of the use of their data. We did not include patients who did not receive chemotherapy because in the 2 institutions in which the patients were treated that we analyzed, patients without chemotherapy were not offered fertility preservation procedures at the time of the study. All medical charts were manually verified from September 2017 to March 2018. The study was approved by the Breast Cancer Study Group of Institut Curie and was conducted in accordance with institutional and ethical rules concerning research on tissue specimens and patients.

The objective of this study was to identify factors associated with discussion of FP in this population, and discussion of FP was used as the primary endpoint.

Under French regulations, written informed consent from patients was not required for this study. This study is a part of the young breast cancer project (YBCP), an institutional project aiming at characterizing BC care pathways in young women. It was approved by the breast cancer group and institutional board (approval 29^th^, April 2019, reference cri-data DATA190136).

### 2.2 Patients

The data collected included age, parity and body mass index (BMI) at diagnosis, date of first consultation at Institut Curie, date of first biopsy showing malignant histological features, date of first chemotherapy, date of surgery, and BRCA status, when available. The date of the first consultation at Institut Curie was taken as the date of diagnosis.

### 2.3 Tumors

We retrieved the following tumor characteristics from the patients’ medical records: clinical T (size) stage and clinical N (nodal) status, immunohistochemical characteristics, such as the detection of estrogen receptors (ER), progesterone receptors (PR), HER2 status, Ki67 and histological grade. Cases were considered estrogen receptor (ER)- or progesterone receptor (PR)-positive (+) if at least 10% of the tumor cells expressed estrogen and/or progesterone receptors (ER/PR), in accordance with the guidelines used in France (16). HER2 expression was assessed by immunohistochemistry, with scoring according to American Society of Clinical Oncology (ASCO)/College of American Pathologists (CAP) guidelines. Scores of 3+ were considered positive, scores of 1+/0 were considered negative (-). Tumors with scores of 2+ were subjected to further testing by FISH. HER2 gene amplification was defined according to ASCO/CAP guidelines (17). Based on immunohistochemical surrogates, pathological breast cancer subtypes were defined as follows: tumors positive for either ER or PR and negative for HER2 were classified as luminal; tumors positive for HER2 were considered HER2-positive BC; tumors negative for ER, PR, and HER2 were considered triple-negative BC (TNBC). Histological grade was determined according to the Elston-Ellis modification of the Scarff-Bloom-Richardson grading system (18).

### 2.4 Treatments

Patients were treated according to national guidelines. Treatments were decided after multidisciplinary consultation meetings considering the characteristics of the patients and prognostic factors. For patients receiving neoadjuvant chemotherapy, surgery was performed four to six weeks after the end of chemotherapy. Trastuzumab was used in an adjuvant and/or neoadjuvant setting for *HER2*-positive breast cancer, in accordance with national guidelines. Most patients received adjuvant radiotherapy. Endocrine therapy (tamoxifen, aromatase inhibitor, and/or GnRH agonists) was prescribed when indicated. Every patient included in our study received chemotherapy (neoadjuvant and/or adjuvant).

### 2.5 Discussion About Fertility Preservation

Discussion about FP (FP discussion) — *i.e.* the delivery of information about the existence of fertility preservation procedure before chemotherapy — was assessed from electronic health records (EHR) as a binary variable (discussion/no discussion), and through a two-way process. Any discussion on damages on fertility induced by chemotherapy counted as “FP Discuss”. Only files with no information on fertility risks were classified as “FP No-Discuss”. We first extracted specific string character patterns by text mining (TM), using specific key words associated with a high likelihood of FP discussion having occurred (“oncofertility”, “IVM”, “frozen oocytes”, “frozen embryos”, “(fertility)”, “ov* fragment preservation”, “ov* cryopreservation”, “ov* cryoconservation”), making it possible to identify the keyword concerned directly in the EHR. This text recognition method was developed and validated on two independent datasets and has been shown to have a better performance than the manual rereading of medical records to identify pregnancies (19). For patients for whom none of the keywords sought was found, we then manually checked all medical consultations between BC diagnosis and chemotherapy, from June to October, 2018.

### 2.6 Physicians

For any consultation with a medical doctor occurring between BC diagnosis and chemotherapy, demographic information about the physician was collected: sex (male *versus* female), age at consultation (junior < 45 years old *versus* senior > 45 years old) and type of specialty (surgeon, oncologist or radiotherapist), together with the rank of healthcare provider (ranging from 1 to 3). Once FP had been discussed with a healthcare provider, subsequent consultations were censored.

### 2.7 Fertility Preservation Procedures

Specific data concerning the procedures were retrieved from the three partner fertility preservation centers in the Parisian region: Jean Verdier Hospital in Bondy, Antoine Beclere Hospital in Clamart and Port Royal Hospital in Paris. We collected the following information: the final choice of the patients or the physician concerning FP procedures, recorded as a binary variable (yes/no), and the method used (oocyte or embryo vitrification after IVM or after controlled ovarian stimulation (COS), cortex cryopreservation).

### 2.8 Statistical Methods

The study population was described in terms of frequencies for qualitative variables, or medians and associated ranges for quantitative variables. For the comparison of continuous variables between groups, Wilcoxon-Mann-Whitney tests were used for groups including fewer than 30 patients, and for variables with multimodal distributions, and Student’s *t* tests were performed otherwise. Associations between categorical variables were assessed in chi-squared tests, or with Fisher’s exact test if at least one category included fewer than three patients. A value of *P* < 0.05 was considered significant. Data were processed and statistical analyses performed with R software version 3.1.2 [www.cran.r-project.org, (R Foundation for Statistical Computing, 2009)].

Data were evaluated using multiple correspondence analysis (MCA). This method involves a multivariate analysis of categorical data and allows joint observation of a vast number of variables. By grouping various characteristics, it attempts to establish a profile capable of suggesting a predisposition to specific situations. Analysis was conducted with the package library (FactoMineR), which performs various mathematical procedures to define the best organization of variables and allocate variables into a four-quadrant plot divided by two axes. Results are interpreted by observation of clusters formed by variables. These clusters represent relations between the variables; the closer they are on the plot, the greater the frequency of their co-occurrence. The two axes separate variables plotted on the left upper quadrant from those in the right lower quadrant and those in the right upper quadrant from those in the left lower quadrant, establishing groups of variables with opposing profiles. It gives a representation of the absolute contribution of each variable according to its distance from the axis, both towards the positive and towards the negative side; the greater the distance, the greater its significance in the interpretation of results.

We used a mixed model combining mixed effects and random effects for the multivariate analysis. The fixed effects influence the mean of the variable of interest (FP discussion) and the random effects influence only the variance of that variable. We used this model based on the assumption that the observations in our database are not independent (i.e. that the occurrence of a FP discussion can be the same depending on the characteristics of the doctors and patients). Thus, the residual variance of the model is partitioned into a between two components: patients and doctors.

## 3 Results

In total,1357 patients were included in the study (**Table 1**). Median age at BC diagnosis was 38.7 years (range: 18-43 years). Most patients had one (21%) or more children (52%) at BC diagnosis, but 27% did not have children. Median tumor size was 30.3 mm, and 58% of the patients had luminal BCs. All patients received chemotherapy (neoadjuvant (45%)/adjuvant (55%) setting). The characteristics of the patients and their tumors differed according to age at BC diagnosis (**Supplementary Table 2**), with a larger number of patients having children (81% *versus* 66%), a larger proportion of luminal tumors (64% *versus* 54%), and a lower likelihood of receiving neoadjuvant as opposed to adjuvant chemotherapy (35% *versus* 52%) in older patients than in younger patients.

**Table 1:** Patient and tumor characteristics (*n*=1357) as a function of the presence or absence of discussion about fertility preservation.

Variable name	Level	Overall	FP discussion	No FP discussion	*p*
	*n*	1357 (100%)	447 (33%)	909 (67%)	
Age (year)	[0 -30)	95 (7%)	72 (76%)	23 (24%)	**<0.001**
	[30 -35)	246 (18%)	173 (70%)	73 (30%)	
	[35 -40)	460 (34%)	162 (35%)	298 (65%)	
	40+	554 (41%)	40 (7%)	514 (93%)	
Age (mean)		38.7 [34.9, 41.6]	34.4 [31.2, 37.2]	40.5 [37.6, 42.3]	**<0.001**
Number of children	0	373 (27%)	231 (62%)	141 (38%)	**<0.001**
	1	279 (21%)	99 (35%)	180 (65%)	
	More than 1	705 (52%)	117 (17%)	588 (83%)	
BMI	<18.5	78 (6%)	29 (37%)	49 (63%)	**0.002**
	18.5-24.9	811 (65%)	302 (37%)	509 (63%)	
	25-29.9	257 (21%)	88 (34%)	168 (66%)	
	>=30	107 (8%)	20 (19%)	87 (81%)	
BMI (mean)		22.6 [20.4, 25.5]	22.3 [20.3, 24.9]	22.8 [20.7, 25.9]	**0.004**
Treatment center	Curie Paris	818 (60%)	287 (35%)	531 (65%)	**0.047**
	Curie St Cloud	538 (40%)	160 (30%)	378 (70%)	
Year of BC diagnosis	2011	167	23 (14%)	144 (86%)	**<0.001**
	2012	190	31 (16%)	159 (84%)	
	2013	186	51 (27%)	135 (73%)	
	2014	224	69 (31%)	155 (69%)	
	2015	221	109 (49%)	112 (51%)	
	2016	216	96 (44%)	120 (56%)	
	2017	151	68 (45%)	83 (55%)	
Hereditary predisposition	No	547 (80%)	240 (44%)	306 (56%)	0.235
	Yes	140 (20%)	70 (50%)	70 (50%)	
Inflammatory BC	No	1338 (99%)	443 (33%)	895 (67%)	0.469
	Yes	18 (1%)	4 (22%)	14 (78%)	
Clinical tumor size (mm)		30.3 (21.7%)	31.5 (20.3%)	29.7 (22.4)	0.148
Clinical T stage (TNM)	T0-T1	588 (44%)	178 (30%)	410 (70%)	**0.052**
	T2	592 (39%)	217 (37%)	375 (63%)	
	T3-T4	166 (12%)	51 (31%)	115 (69%)	
Clinical N stage (TNM)	N0	854 (63%)	281 (33%)	573 (67%)	0.859
	N1-N2-N3	492 (36%)	165 (34%)	327 (66%)	
SBR grade	Grade I	58 (4%)	16 (28%)	42 (72%)	**0.006**
	Grade II	528 (39%)	150 (28%)	378 (72%)	
	Grade III	760 (57%)	278 (37%)	482 (63%)	
BC subtype	Luminal	702 (58%)	208 (30%)	494 (70%)	**0.021**
	TNBC	241 (20%)	92 (38%)	148 (62%)	
	HER2^+^/HR^+^	193 (16%)	75 (39%)	118 (61%)	
	HER2^+^/HR^-^	81 (6%)	28 (35%)	53 (65%)	
Histological type	NST	1265 (93%)	426 (34%)	839 (66%)	**0.014**
	Lobular	54 (4%)	8 (15%)	46 (85%)	
	Others	36 (3%)	13 (36%)	23 (64%)	
Chemotherapy setting	Adjuvant	744 (55%)	201 (27%)	543 (73%)	**<0.001**
	NAC	611 (45%)	245 (40%)	366 (60%)	
FP procedure	No	1095 (81%)	188 (17%)	906 (83%)	**<0.001**
	Yes	262 (19%)	259 (99%)	3 (1%)	
LHRH Analogs	No	1340 (99%)	242 (18%)	1098 (82%)	**<0.001**
	Yes	17 (1%)	17 (100%)	0 (0%)	

Values in bold are the significant values.

### 3.1 Factors Associated With Fertility Discussion

Some mention of FP discussion was found in the EHRs of 447 (33%) of the 1357 patients, whereas no such mention was not found in the EHRs of 909 patients (67%).

#### 3.1.1 Patient-Related Factors

**Figure 1:**
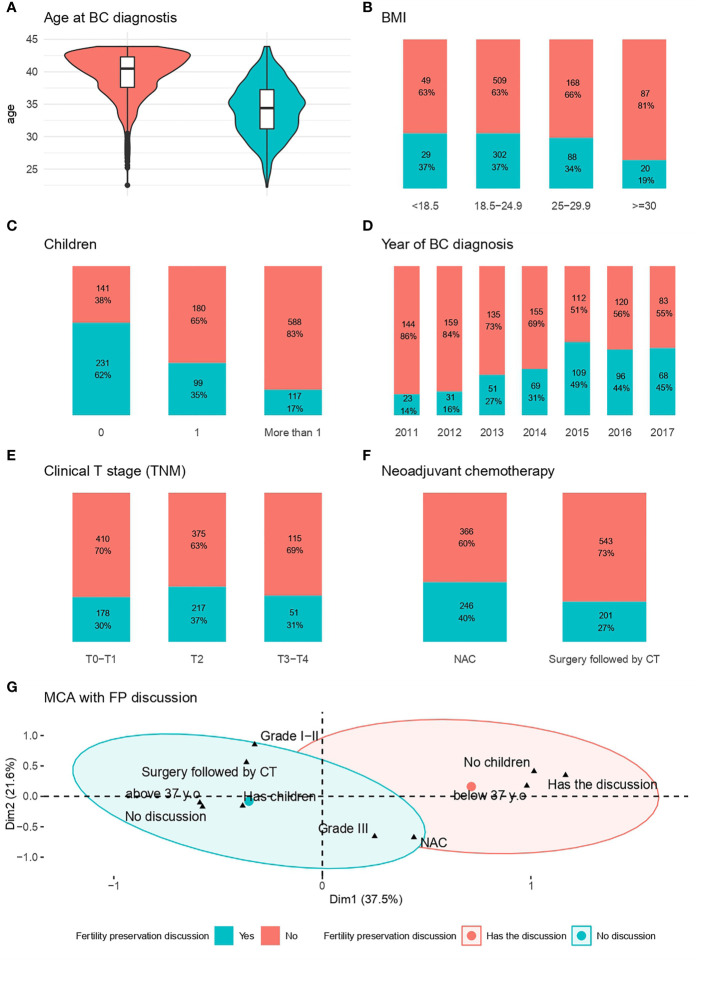
Factors associated with the likelihood of FP Discussion. **(A)** Age at BC diagnosis; **(B)**: BMI; **(C)** Patient with children at the time of diagnosis; **(D)** Year of diagnosis; **(E)** Clinical stage (TNM); **(F)** Neoadjuvant chemotherapy; **(G)** MCA for fertility preservation discussion*. *The red ellipse represents the concentration of people who had no discussion about fertility preservation, whereas the blue ellipse represents the concentration of people who discussed fertility preservation with a physician.

FP discussion was significantly associated with a younger age at BC diagnosis (median age 34 years *versus* 40 years, *p*<0.001) (**Figure 1A**), and were less likely to occur for obese patients (**Figure 1B**). Her frequency decreased with increasing numbers of children (**Figure 1C**) and increased over the time period of the cohort, reaching a plateau at about 45% after 2015 (**Figure 1D**). Clinical stage (*p*=0.05) and neoadjuvant chemotherapy (*p*<0.001) were also significantly associated with FP discussion (**Figures 1E, F** respectively). Multiple component analysis identified two groups of patients and characteristics associated with fertility discussion (red ellipse: patients aged 37 years or older, with children at diagnosis, for whom there was no FP discussion; and blue ellipse: patients below the age of 37, with no children at diagnosis, for whom FP discussion occurred) (**Figure 1G**).

#### 3.1.2 Doctor- and Center-Related Factors

In total, 2468 pre-chemotherapy consultations were retrieved from the EHRs (with surgeons *n*=1280; medical oncologists *n*=1073, and radiotherapy oncologists, *n*=115) (**Table 2**).

**Table 2:** Likelihood of FP discussion according to physician characteristics and center (*n*=2468).

Variable name	Level	Overall	FP Discussion	No FP Discussion	p
	*n*	2468	447	2021	
Specialty	Oncologist	1073 (43%)	150 (14%)	923 (86%)	**<0.001**
	Radiotherapy oncologist	115 (5%)	12 (10%)	103 (90%)	
	Surgeon	1280 (52%)	285 (22%)	995 (78%)	
Age	Junior	937 (38%)	193 (21%)	744 (79%)	**0.017**
	Senior	1521 (62%)	254 (17%)	1267 (83%)	
Sex	Female	1292 (52%)	274 (21%)	1018 (79%)	**<0.001**
	Male	1169 (48%)	173 (15%)	996 (85%)	
Treatment center	Center 1	1454 (59%)	287 (20%)	1167 (80%)	0.143
	Center 2	1014 (41%)	160 (16%)	854 (84%)	

Values in bold are the significant values.

FP discussion was more frequently mentioned during the first pre-chemotherapy consultation, than during the following visits (discussed with the first practitioner n=336; second n=92; third n=19).

**Figure 2:**
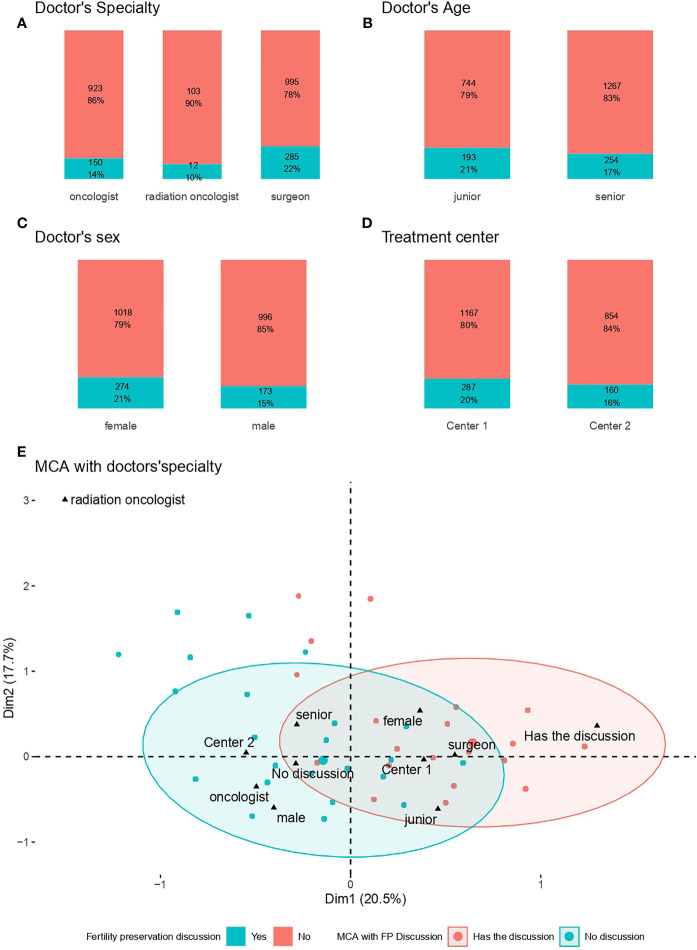
Factors associated with fertility preservation Discussion. **(A)** Doctors’ specialty; **(B)** Doctors’ age; **(C)** Doctors’ sex; **(D)** Treatment Center; **(E)** MCA with FP discussion*. **(E)** The red ellipse represents the concentration of patients who did have discussion about fertility preservation, whereas the blue ellipse represents the concentration of patients who discussed fertility preservation with a physician.

Doctors’ specialty was significantly associated with the likelihood of FP discussion. Surgeons were more likely to discuss FP with patients (22%) than medical oncologists (14%) and radiation oncologists (10%) (**Figure 2A**). Doctors’ age (**Figure 2B**) and sex (**Figure 2C**) were also significantly associated with FP discussion: junior doctors (21%) and female doctors (21%) were slightly more likely to discuss FP than senior doctors (17%) and male doctors (15%), respectively. The site where patients received their treatment (center 1 *versus* center 2) (**Figure 2D**) was not significantly associated with the likelihood of FP discussion (*p*=0.14).

Multiple correspondence analysis identified two groups of physicians and characteristics associated with FP discussion (red ellipse: oncologists and male physicians not discussing FP with patients; blue ellipse: surgeons and female physicians discussing FP with patients) (**Figure 2E**).

#### 3.1.3 Factors Associated With FP Discussion in Multivariate Analysis

After multivariate analysis with the mixed model, fertility discussion was significantly associated with younger age, number of children before BC diagnosis, physicians’ gender and physicians’ specialty (**Table 3**).

**Table 3:** Factors associated with FP discussion in multivariate analysis (mixed model).

Variable name	Level	OR (IC 95%)	*p*
**Patient characteristics**			
Age (year)	[0 -30)	1.00	
	[30 -35)	1.24 (0.77-1.98)	0.375
	[35 -40)	0.38 (0.24 – 0.60)	**p<0.001**
	40+	0.05 (0.03 – 0.09)	**p<0.001**
Number of children	0	1.00	
	0 - 1	0.39 (0.27 – 0.54)	**p<0.001**
	More than 1	0.17 (0.12 – 0.23)	**p<0.001**
**Tumor characteristics**			
SBR grade	Grade I	1.00	
	Grade II	0.78 (0.39 – 1.59)	0.498
	Grade III	0.76 (0.38 – 1.53)	0.449
Neoadjuvant chemotherapy	No	1.00	
	Yes	1.15 (0.88 – 1.50)	0.298
**Physician characteristics**			
Sex	Female	1.00	
	Male	0.59 (0.35 – 0.99)	**0.048**
Age	Junior	1.00	
	Senior	0.77 (0.46 – 1.31)	0.336
Specialty	Surgeon	1.00	
	Radiotherapy oncologist	0.22 (0.07 – 0.64)	**0.006**
	Oncologist	0.78 (0.46 – 1.32)	0.352

Values in bold are the significant values.

### 3.2 Factors Associated With the Performance of Fertility Preservation Procedures (FPPs)

FP procedures were performed in 262 of the 1357 patients (19%). Seventeen patients received treatment with LHRH analogs. The main factor associated with the occurrence of FPPs was the occurrence of FP discussion (only three patients underwent FPPs without prior FP discussion).

**Figure 3:**
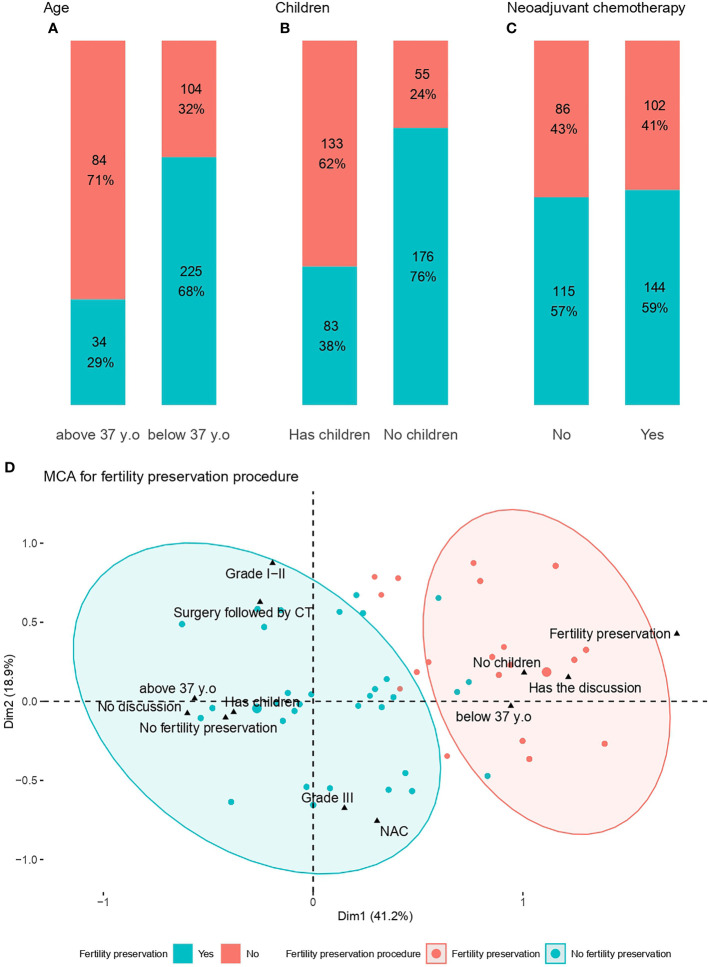
Factors associated with fertility preservation procedures. **(A)** Age at diagnosis; **(B)** Children; **(C)** Neoadjuvant chemotherapy; **(D)** MCA for fertility preservation procedures*. *The red ellipse represents the concentration of patients who did not undergo fertility preservation procedures, whereas the blue ellipse represents the concentration of patients who underwent fertility preservation procedures.

Out of 447 patients who had a FP discussion, 259 patients (58%) had a FP procedure and 188 (42%) didn’t have (**Table 4**). The factors significantly associated with the realization of a FPP in FP discussion group were age (**Figure 3A**), and previous children (**Figure 3B**). The type of chemotherapy was not associated with PPF (**Figure 3C**). The MCA clustered patients into two distinct groups, with FP discussion, already having children, and age as the major factors explaining the performance of a FPP (**Figure 3D**).

**Table 4:** Performance of fertility preservation procedures (FPPs) as a function of patient with FP discussion characteristics (n = 447).

Variable name	Level	Overall	FP Procedure	No FP Procedure	*p*
	*n*	447	259 (58%)	188 (42%)	
Age (year)	[0 -30)	72	63 (88%)	9 (12%)	**<0.001**
	[30 -35)	173	118 (68%)	55 (32%)	
	[35 -40)	162	75 (46%)	87 (54%)	
	40+	40	3 (8%)	37 (92%)	
Age (mean)		34.2 (4.1)	32.7 (3.7)	36.3 (3.7)	**<0.001**
Number of children	0	231	176 (76%)	55 (24%)	**<0.001**
	1	99	51 (52%)	48 (48%)	
	More than 1	117	32 (27%)	85 (73%)	
BMI	<18.5	29	15 (52%)	14 (48%)	0.543
	18.5-24.9	302	181 (60%)	121 (40%)	
	25-29.9	88	46 (52%)	42 (48%)	
	>=30	20	12 (60%)	8 (40%)	
BMI (mean)		22.3 [20.3, 24.9]	22.0 [20.3, 24.5]	22.6 [20.4, 25.1]	0.327
Treatment center	Curie Paris	287	167 (58%)	120 (42%)	0.967
	Curie St Cloud	160	92 (57%)	68 (42%)	
Year of BC diagnosis	2011	23	10 (43%)	13 (57%)	0.063
	2012	31	22 (71%)	9 (29%)	
	2013	51	36 (71%)	15 (29%)	
	2014	69	33 (48%)	36 (52%)	
	2015	109	62 (57%)	47 (43%)	
	2016	96	60 (62%)	36 (38%)	
	2017	68	36 (53%)	32 (47%)	
Hereditary predisposition	No	240	156 (65%)	84 (35%)	1.000
	Yes	70	46 (66%)	24 (34%)	
Clinical tumor size (mm)		31.5 (20.3)	32.2 (20.2%)	30.6 (20.4)	0.428
Clinical T stage (TNM)	T0-T1	178	94 (53%)	84 (47%)	0.168
	T2	217	135 (62%)	82 (38%)	
	T3-T4	51	29 (57%)	22 (43%)	
Clinical N stage (TNM)	N0	281	163 (58%)	118 (42%)	1.000
	N1-N2-N3	165	95 (58%)	70 (42%)	
SBR grade	Grade I	16	11 (69%)	5 (31%)	0.212
	Grade II	150	94 (63%)	56 (37%)	
	Grade III	278	153 (55%)	125 (45%)	
BC subtype	Luminal	208	120 (58%)	88 (42%)	0.609
	TNBC	92	54 (59%)	38 (41%)	
	HER2^+^/HR^+^	75	48 (64%)	27 (36%)	
	HER2^+^/HR^-^	28	14 (50%)	14 (50%)	
Histological type	NST	426	246 (58%)	180 (42%)	0.591
	Lobular	8	6 (75%)	2 (25%)	
	Others	13	7 (54%)	6 (46%)	
Neoadjuvant chemotherapy	No	201	115 (57%)	86 (43%)	0.853
	Yes	246	144 (59%)	102 (41%)	

Values in bold are the significant values.

Most patients (*n*=175) underwent IVM, and one third (*n*=84) had at least one COS. The factors associated with the type of FPP (**Supplementary Table 3**) were mostly related to the chemotherapy setting with COS used in a neoadjuvant setting in only six out of 146 patients.

## 4 Discussion

This large, real-life study found that the rates of the discussion of fertility preservation (FP) were low (33%) in a consecutive series of 1,357 female breast cancer patients exposed to gonadotoxic chemotherapy. Furthermore, we discussed the correlation between FP discussion and the characteristics of patients/tumors and healthcare practitioners. We found that younger age, number of children before breast cancer diagnosis, physicians’ gender and physician’s specialty were independent predictors of FP discussion. We also found an increased likelihood of FP discussion over time. In general, the findings in the present study support the above-mentioned conclusion. The results of our study confirm and reinforce previous findings from the literature.

One of its key findings is that FP discussion was mentioned in only one third of EHRs. These rates lie in the lower part of the range of published values, which generally range from 30 to 70% (9, 12, 20). There are several possible reasons for these low rates. First, we included patients up to 43 years old, and patients 40 y.o. or above represented 41% of the cohort. When focusing only in the subpopulation of patients below 40, the discussion rate increased to 51%. Second, this cohort study began in 2011, a time at which FP had yet to emerge as a major issue. In addition, since 2011, vitrification can be performed in France, which improves the results of embryo and especially oocyte freezing. The improvement in practices over time indicates an increase in the awareness of healthcare practitioners. A plateau was nevertheless reached in 2015, and the proportion of patients for whom FP was discussed never exceeded 50%. This result is consistent with previous studies (10) indicating a significant, but nevertheless incomplete, improvement in practices. Another possible reason is that the Institut Curie is a specialist cancer center focusing purely on oncology care. Thus, unlike multispecialty clinics, it does not have its own gynecology or reproductive biology department.

Our findings confirm that several patient-related factors are associated with the likelihood of FP discussion, as summarized in **Supplementary Table 4** (BC) and **Supplementary Table 5** (all cancer types). Earlier age at diagnosis was significantly associated with a greater likelihood of FP discussion (8, 9). The frequency of FP discussion was 35% in women aged 35 years or older, falling to 7% in women over the age of 40 years. The mixed model of our study confirms the impact of age on FP discussion. Age at diagnosis is a well-known, important factor associated with FP discussion, and this association has been found to be significant in most studies. This finding is nevertheless a matter of concern, because the proportion of women diagnosed with BC increases steadily with age, and most “young” BC patients are already at least 37 years old at BC diagnosis. There are currently no guidelines specifying that such discussion is dispensable for women over the age of 37 years. For the use of vitrified oocytes, French guidelines consider that it is imperative to take into account obstetrical morbidity, which increase with age (after 45 years, pregnancy is at high risk of complications and even more after the age of 50 years) (21). Not all patients will be eligible for FP, but it is essential to have a discussion with them about their options and about post-cancer infertility.

Consistent with another study (20), we found that nulliparity was significantly associated with FP discussion. Such discussion took place for only 17% for patients who already had more than one child at diagnosis. Thewes et al. reported that about 70% of 228 BC patients under the age of 45 years wished to have a child after their treatment was completed (8). Marklund et al. (22), analyzed a cohort of 1275 BC patients and found that 171 patients (33%) had a live birth after the end of treatment, and that 63% of these patients already had at least one child at diagnosis.

In our study, no factor related to BC disease (clinical T stage, lymph node status, SBR grade, BC subtype, histological type) was found to be significantly associated with the likelihood of FP discussion. Conflicting results have been reported (8), but several studies (10) have suggested that early-stage disease is more frequently associated with FP discussion. We did not include bilateral breast cancer which makes more complex statistical analyses as it requires the use of multilevel models, and it causes difficulties in attributing relapse to one or to the other side. Furthermore, it is very unlikely that the results are biased because synchronous bilateral breast cancers represent 1-3% (23). In terms of treatment, FP was more frequently discussed in the group of patients receiving neoadjuvant chemotherapy than in patients receiving adjuvant chemotherapy, but this was highly probably due to age acting as a confounding factor, because it was very significantly associated with the chemotherapy setting. We did not include patients who did not receive chemotherapy, but we must highlight that the patients who did not receive chemotherapy represent a very minority in this age group (15 to 20%). We found no impact of type of chemotherapy, hormone therapy, or radiation therapy, consistent with the findings of other studies (9, 24). However, FP is an important subject in this context, because hormone therapy can delay pregnancy plans by at least two to three years.

Several practitioner-related factors were associated with the likelihood of FP discussion, including specialty in particular. Surgeons were the most likely to discuss FP with their patients, followed by medical oncologists and then radiotherapists. We also identified the sex and age of the medical practitioner as significantly associated with the likelihood of FP discussion. Korkidakis et al. (20) analyzed a cohort of 4,452 breast cancer patients aged 15-39 years before chemotherapy treatment and obtained similar results, with female physicians and surgeons the most likely to discuss FP with their patients. Covelli et al. (25) investigated the barriers to physicians discussing fertility and found that physicians often assigned responsibility for fertility counselling to other clinicians and felt a lack of confidence in their ability to initiate FP discussion. Patel et al. (26) found that multi-specialty clinics had lower rates of FP counseling concerning fertility risk than single-specialty clinics. One possible reason for this difference may be a lack of clear designation of the doctor responsible for discussing the infertility risk associated with chemotherapy. Multicenter studies have identified regional disparities in information about FP, and differences between oncology centers (10), but we found no significant differences between the cancer centers in our study.

Finally, we confirm the crucial importance of FP discussion for favoring the performance of FPPs. Only three of the 262 patients who underwent PF procedures had not previously discussed FP with their doctors. Our data therefore indicate that a lack of discussion about FP during in-house consultations severely impedes patient choice as to whether to undergo FPPs. However, almost one third (188/447) of the patients who received information about FP chose not to undergo FPPs, or were not eligible for the procedures. We found the same factors associated with the FP procedure in the group of patients who had a FP discussion: age and parity. Previous studies analyzing annual income or health insurance as possible factors influencing discussion about FP found no association with these factors (9), which can be ruled out in our study because all the patients were covered by a universal social security system guaranteeing the full reimbursement of FP fees, up to 43 years.

Our study has several strengths, in particular, the inclusion of a large number of patients and doctors, allowing an analysis of a multitude of variables. However, it also has limitations, such as its retrospective nature, in particular. Information on the FP discussion was retrospectively obtained from the patient’s electronic health record. Since some doctors may not record their discussion with patients about FP in the electronic health record system, results from this study may underestimate the rates of FP discussion. The healthcare providers play an important role in the discussion of fertility preservation. However, less than 50% of the patients had FP with their doctors and only 19% of the patients had FP procedures. More characteristics of the healthcare providers are recommended to be analyzed and discussed, such as their knowledge about FP procedures, or how much time spent for each communication on FP with patients would be of major interest to further understand determinants associated with physician’s related barriers and facilitators.

This work has several clinical implications and identifies areas in which there is room for improvement. It highlights a patient population with unmet needs regarding information on FP (patients in their late 30s who already have children). It also calls for better training for healthcare providers to raise awareness on this topic, particularly among male doctors, through seminars (27), joint training with reproductive medicine experts (28), or the development of FP networks (29).

Prestructured fields in the EHR may be pertinent tools for preventing omissions and could provide an alert in real time, prompting such discussion. Alerts of this type have already proved effective for preventing drug interactions and are currently used in this context (30, 31). A similar reminder could be issued for all women of childbearing age receiving gonadotoxic treatment, to improve oncofertility practices in cancer care. To clear the delineation of who is responsible for discussing the infertility risk associated with chemotherapy, the discussion could be done at the first consultation, which would facilitate a better systematization of the information. Finally, providing patients with information directly, *via* posters or flyers in waiting rooms, patient advocacy, and communities could help to increase the proportion of patients who are informed and empowered, and able to decide independently whether or not they wish to undergo FPPs if it is possible, before receiving gonadotoxic treatment.

## Data Availability Statement

The original contributions presented in the study are included in the article/**Supplementary Material**. Further inquiries can be directed to the corresponding author.

## Author Contributions

Conceptualization, AH, AT, FR, and A-SH. Methodology, FC and A-SH. Software, JB, A-SH. Validation, AH, AT, VC, CaS, A-SH, and FR. Formal analysis, A-SH. Investigation, AH, AT, VC, J-YP, and J-GF. Data curation, AH, AT, CaS, and A-SH. Writing—original draft preparation, AH and A-SH. Writing—review and editing, AH, AT, CaS, EL, BG, AT, FC, EDu, EDa, J-YP, J-GF, FR, and A-SH. All authors contributed to the article and approved the submitted version.

## Funding

The authors declare that this study is a part of The Young Breast Cancer Project. The Young Breast Cancer Project was funded by Monoprix. The funder was not involved in the study design, collection, analysis, interpretation of data, the writing of this article or the decision to submit it for publication.

## Conflict of Interest

The authors declare that the research was conducted in the absence of any commercial or financial relationships that could be construed as a potential conflict of interest.

## Publisher’s Note

All claims expressed in this article are solely those of the authors and do not necessarily represent those of their affiliated organizations, or those of the publisher, the editors and the reviewers. Any product that may be evaluated in this article, or claim that may be made by its manufacturer, is not guaranteed or endorsed by the publisher.
