# Supplementary material for: Factors Associated With the Discussion of Fertility Preservation in a Cohort of 1,357 Young Breast Cancer Patients Receiving Chemotherapy
Source: Front Oncol. 2021 Sep 28;11:701620. doi: 10.3389/fonc.2021.701620 (PMC8507557; doi:10.3389/fonc.2021.701620)
Supplement: Supplementary file 1 [file DataSheet_1.docx]

Supplementary Material

Suppl. Table 1 - Flow Chart

Suppl. Table 2 - Patients Characteristics as a function of Age

| **Variable name** | **level** | **Overall** | **[0 -40)** | **40+** | ***p*** |
| --- | --- | --- | --- | --- | --- |
|  | *n* | 1357 | 801 | 554 |  |
| Number of children | 0 | 373 (27) | 268 (33) | 103 (19) | **<0.001** |
|  | 1 | 279 (21) | 178 (22) | 101 (18) |  |
|  | More than 1 | 705 (52) | 355 (44) | 350 (63) |  |
| BMI | <18.5 | 78 (6) | 50 (7) | 28 (6) | 0.299 |
|  | 18.5-24.9 | 811 (65) | 494 (66) | 316 (63) |  |
|  | 25-29.9 | 257 (21) | 143 (19) | 113 (22) |  |
|  | >=30 | 107 (9) | 59 (8) | 48 (10) |  |
| BMI (mean) | | 22.6 [20.4, 25.5] | 22.4 [20.3, 25.2] | 22.8 [20.8, 26.0] | **0.005** |
| Treatment center | Curie Paris | 818 (60) | 485 (61) | 332 (60) | 0.862 |
|  | Curie St Cloud | 538 (40) | 316 (39) | 222 (40) |  |
| Genetic analysis | No | 656 (48) | 265 (33) | 390 (70) | **<0.001** |
|  | Yes | 701 (52) | 536 (67) | 164 (30) |  |
| Inflammatory BC | No | 1338 (99) | 788 (98) | 549 (99) | 0.369 |
|  | Yes | 18 (1) | 13 (2) | 5 (1) |  |
| Clinical Tumor size (mm) | | 30.3 (21.7) | 32.6 (21.5) | 27.0 (21.6) | **<0.001** |
| Clinical T stage (TNM) | T0-T1 | 588 (44) | 304 (38) | 283 (51) | **<0.001** |
|  | T2 | 592 (44) | 382 (48) | 210 (38) |  |
|  | T3-T4 | 166 (12) | 108 (14) | 58 (11) |  |
| Clinical N stage (TNM) | N0 | 854 (63) | 493 (62) | 360 (65) | 0.247 |
|  | N1-N2-N3 | 492 (37) | 301 (38) | 191 (35) |  |
| SBR grade | Grade I | 58 (4) | 27 (3) | 31 (6) | **<0.001** |
|  | Grade II | 528 (39) | 279 (35) | 249 (45) |  |
|  | Grade III | 760 (56) | 487 (61) | 272 (49) |  |
| BC subtype | Luminal | 702 (58) | 383 (54) | 318 (64) | **0.003** |
|  | TNBC | 241 (20) | 163 (23) | 77 (15) |  |
|  | HER2^+^/HR^+^ | 193 (16) | 120 (17) | 73 (15) |  |
|  | HER2^+^/HR^-^ | 81 (7) | 49 (7) | 32 (6) |  |
| Histological type | NST | 1265 (93) | 762 (95) | 502 (91) | **0.001** |
|  | Lobular | 54 (4) | 19 (2) | 35 (6) |  |
|  | Others | 36 (3) | 20 (2) | 16 (3) |  |
| Neoadjuvant chemotherapy | No | 744 (55) | 382 (48) | 361 (65) | **<0.001** |
|  | Yes | 612 (45) | 419 (52) | 193 (35) |  |
| FP discussion | No | 909 (67) | 394 (49) | 514 (93) | **<0.001** |
|  | Yes | 447 (33) | 407 (51) | 40 (7) |  |

Suppl. Table 3 - Patients Characteristics as a function of methods of fertility preservation

| **Variable name** | **level** | **Overall** | **IVM** | **At least one COS** | ***p*** |
| --- | --- | --- | --- | --- | --- |
|  | *n* | 266 | 175 | 84 |  |
| Age (year) | [0 -30) | 63 (24) | 45 (26) | 16 (19) | 0.185 |
|  | [30 -35) | 121 (45) | 83 (47) | 35 (42) |  |
|  | [35 -40) | 78 (29) | 44 (25) | 32 (38) |  |
|  | 40+ | 4 (2) | 3 (2) | 1 (1) |  |
| Age (mean) | | 32.8 (3.7) | 32.4 (3.8) | 33.5 (3.7) | 0.042 |
| Number of children | 0 | 181 (68) | 120 (69) | 56 (67) | 0.921 |
|  | 1 | 53 (20) | 35 (20) | 17 (20) |  |
|  | More than 1 | 32 (12) | 20 (11) | 11 (13) |  |
| BMI | <18.5 | 16 (6) | 5 (3) | 10 (12) | 0.026 |
|  | 18.5-24.9 | 185 (71) | 121 (71) | 58 (70) |  |
|  | 25-29.9 | 47 (18) | 35 (21) | 12 (14) |  |
|  | >=30 | 12 (5) | 9 (5) | 3 (4) |  |
| BMI (mean) | | 22.0 [20.3, 24.4] | 22.5 [20.3, 25.1] | 21.5 [20.1, 24.3] | 0.074 |
| Treatment center | Curie Paris | 171 (64) | 115 (66) | 53 (63) | 0.784 |
|  | Curie St Cloud | 95 (36) | 60 (34) | 31 (37) |  |
| Genetic analysis | No | 56 (21) | 34 (19) | 21 (25) | 0.388 |
|  | Yes | 210 (79) | 141 (81) | 63 (75) |  |
| Inflammatory BC | No | 264 (99) | 173 (99) | 84 (100) | 0.822 |
|  | Yes | 2 (1) | 2 (1) | 0 (0) |  |
| Clinical tumor size (mm) | | 31.7 (20.1) | 36.7 (21.1) | 21.7 (13.9) | **<0.001** |
| Clinical T stage (TNM) | T0-T1 | 101 (38) | 42 (24) | 56 (67) | **<0.001** |
|  | T2 | 135 (51) | 109 (62) | 22 (27) |  |
|  | T3-T4 | 29 (11) | 24 (14) | 5 (6) |  |
| Clinical N stage (TNM) | N0 | 169 (64) | 101 (58) | 62 (75) | **0.012** |
|  | N1-N2-N3 | 96 (36) | 74 (42) | 21 (25) |  |
| SBR grade | Grade I | 11 (4) | 8 (5) | 3 (4) | 0.772 |
|  | Grade II | 97 (37) | 63 (36) | 34 (40) |  |
|  | Grade III | 157 (59) | 103 (59) | 47 (56) |  |
| BC subtype | Luminal | 121 (50) | 75 (47) | 45 (59) | 0.388 |
|  | TNBC | 55 (23) | 37 (23) | 14 (18) |  |
|  | HER2^+^/HR^+^ | 50 (21) | 37 (23) | 13 (17) |  |
|  | HER2^+^/HR^-^ | 14 (6) | 10 (6) | 4 (5) |  |
| Histological type | NST | 252 (95) | 169 (97) | 77 (92) | 0.230 |
|  | Lobular | 6 (2) | 3 (2) | 3 (4) |  |
|  | Others | 8 (3) | 3 (2) | 4 (5) |  |
| Neoadjuvant chemotherapy | No | 120 (45) | 39 (22) | 78 (93) | **<0.001** |
|  | Yes | 146 (55) | 136 (78) | 6 (7) |  |
| FP discussion | No | 3 (1) | 2 (1) | 1 (1) | 1.000 |
|  | Yes | 263 (99) | 173 (99) | 83 (99) |  |

Suppl. Table 4 - Summary of the characteristics and results of the different studies evaluating the factors associated with the discussion of fertility preservation in patients receiving gonadotoxic treatments for breast cancer

| Study | Author | Year | Journal | n = | Type of study | Source information about FP discussion | Year of Diagnosis | Population | FP Discussion | FPP | Patients and Tumor Characteristics Factors | | | | | | | | | Physicians Factors | | |
| --- | --- | --- | --- | --- | --- | --- | --- | --- | --- | --- | --- | --- | --- | --- | --- | --- | --- | --- | --- | --- | --- | --- |
|  |  |  |  |  |  |  |  |  |  |  | Age | Parity | Disease | NAC/AC | HT | Year | Heredit | Centre | Marital St | Age | Gender | Special |
| This Study | Hours | 2021 | NA | 1357 | Retrospective cohort study | Text Mining and EHR reviewed | 2011-2017 | Women aged 18 to 43 years **Breast** cancer Chemotherapy | 33% | 19% | **yes** | **yes** | **no** | **yes** | **no** | **yes** | **no** | **no** | NA | **no** | **yes** | **yes** |
| Discussions Regarding Reproductive (…) | Duffy | 2005 | JCO | 107 | Randomized controlled trial | Phone Interview and E-mail | 1996-1999 | Women aged 45 years or younger  Previous **breast** cancer Beginning their first course of Chemotherapy | 34% | NA | **yes** | **no** | **no** | NA | **no** | NA | NA | NA | **no** | NA | NA | NA |
| Fertility and menopause related information (…) | Thewes | 2005 | JCO | 228 | Retrospective cohort study | Questionnaire | NA | Women aged 18 to 40 years  **Breast** cancer  Chemotherapy or/and endocrine therapy | 71% | NA | **yes** | **yes** | **no** | **no** | **no** | NA | NA | NA | **yes** | NA | NA | NA |
| Determinants of access to fertility preservation (…) | Lee | 2011 | Fertil Steril | 314 | Retrospective cohort study | Early referral or delayed referral | 1999-2009 | Women under the age of 45 years **Breast** cancer Chemotherapy and/or radiotherapy | 69.4% (early group) | NA | **yes** | **no** | **yes** | NA | NA | **yes** | **yes** | **yes** | NA | NA | NA | NA |
| Satisfaction with fertlity and sexuality related (…) | Ben Charif | 2015 | BMC Cancer | 319 | Longitudinal prospective study | Questionnaire | 2009-2014 | Women aged 18 and 40 yearsto**Breast** cancer | 53% | NA | **no** | **no** | NA | **no** | **no** | NA | **yes** | **yes** | **no** | NA | NA | NA |
| Patterns of referras for fertility preservation (…) | Korkidakis | 2019 | Journal of AYA oncology | 4452 | Retrospective cohort study | FP referral (infertility diagnostic code) | 2000-2017 | Women aged 15 to 39 years **Breast** cancer Chemotherapy | 4% | NA | **yes** | **yes** | **yes** | NA | NA | **yes** | NA | NA | NA | **no** | **yes** | **yes** |

Abbreviation: NA: not applicable/not available, NAC/AC: neoadjuvant/adjuvant chemotherapy, HT: hormonotherapy, Heredit: hereditary predisposition, Marital St: marital status, FP: fertility preservation, FPP: fertility preservation procedure, Special: Speciality

“yes”: statistically significant

“no”: no statistically significant

Suppl. Table 5 - Summary of the characteristics and results of the different studies that have evaluated the factors associated with a discussion of fertility preservation in patients receiving gonadotoxic treatments in All Type of Cancer

| ^Study^ | ^Author^ | ^Year^ | ^Journal^ | *^n^* ^=^ | ^Type of study^ | ^Source information of FP discussion^ | ^Year of Diagnosis^ | ^Population^ | ^FP Discussion^ | ^FPP^ | ^Patients and Tumor Characteristics Factors^ | | | | | | | | | ^Praticians Factors^ | | | | |
| --- | --- | --- | --- | --- | --- | --- | --- | --- | --- | --- | --- | --- | --- | --- | --- | --- | --- | --- | --- | --- | --- | --- | --- | --- |
|  |  |  |  |  |  |  |  |  |  |  | ^Age^ | ^Parity^ | ^Disease^ | ^NAC/AC^ | ^HT^ | ^Year^ | ^Heredit^ | ^Centre^ | ^Marital St^ | | ^Age^ | ^Gender^ | ^Speciality^ |  |
| ^Racial, Socioeconomic, and Demographic Disparities (…)^ | ^Letourneau^ | ^2012^ | ^Cancer^ | ^918^ | ^Analytic cross-sectional study, multicentric^ | ^E-mail, letter and phone call^ | ^1993-2007^ | ^Women between 18 to 40 years Cancers: leukemia, Hodgkin and non-Hodgkin lymphoma, gastrointestinal cancer  Gonadotoxic treatment^ | ^61%^ | ^4%^ | **^yes^** | **^no^** | ^NA^ | **^no^** | **^no^** | ^NA^ | ^NA^ | ^NA^ | **^no^** | | ^NA^ | ^NA^ | ^NA^ |  |
| ^Fertility status perception, fertility (…)^ | ^Jegaden^ | ^2018^ | ^Future Science^ | ^548^ | ^Retrospective cohort study^ | ^Questionnaire: 2 years and 5 years after diagnosis^ | ^2010^ | ^Men and women aged between 18 and 40 years (79.5% women = 427) All cancers (46,9% breast)^ | ^32.6% (women)^ | ^2.8% (women)^ | **^yes^** | **^yes^** | ^NA^ | **^yes^** | ^no^ | ^NA^ | ^NA^ | ^NA^ | **^no^** | | ^NA^ | ^NA^ | ^NA^ |  |
| ^Infertility induced by cancer treatment (…)^ | ^Mancini^ | ^2008^ | ^Fertil^  ^Steril^ | ^282 (women)^ | ^Retrospective cross-sectional study^ | ^Telephone interview^ | ^2002^ | ^Women aged 18 to 44 years  All cancers, 2 years after diagnosis (46% breast)^ | ^70.2%^ | ^63.1%^ | **^yes^** | **^no^** | **^no^** | **^no^** | **^yes^** | ^NA^ | ^NA^ | ^NA^ | **^no^** | | ^NA^ | ^NA^ | ^NA^ |  |
| ^Sex differences in dertility-related dnformation (…)^ | ^Armuand^ | ^2012^ | ^JCO^ | ^328 (women)^ | ^Retrospective cohort study^ | ^Questionnaire^ | ^2003-2007^ | ^Women aged 18 to 45 years at diagnosis  Cancers: lymphoma, acute leukemia, ovarian cancer, BC Treatment with chemotherapy^ | ^14% (women)^ | ^NA^ | **^yes^** | **^yes^** | ^NA^ | ^NA^ | ^NA^ | ^NA^ | ^NA^ | ^NA^ | ^NA^ | | ^NA^ | ^NA^ | ^NA^ |  |
| ^Which female cancer patients fail to receive (…)^ | ^Chin^ | ^2016^ | ^Fertil Steril^ | ^1116^ | ^Population-based cohort study descriptive^ | ^Telephone interview^ | ^1990-2009^ | ^Women aged 20 to 35 years  All cancers, 2 years after diagnosis^ | ^59%^ | ^NA^ | **^no^** | **^yes^** | ^NA^ | **^yes^** | ^NA^ | **^yes^** | ^NA^ | ^NA^ | **^yes^** | | ^NA^ | ^NA^ | ^NA^ |  |
| ^Disparities in counseling female cancer (…)^ | ^Lawson^ | ^2017^ | ^JWH^ | ^259^ | ^Retrospective cohort study, unicentric^ | ^EMR reviewed^ | ^2009-2013^ | ^Women aged 18 to 45 years  Cancers: breast, gynecological, hematological cancer  Gonadotoxic chemotherapy^ | ^61.8%^ | ^NA^ | **^yes^** | ^NA^ | ^NA^ | ^NA^ | ^NA^ | **^yes^** | ^NA^ | ^NA^ | **^yes^** | | ^NA^ | ^NA^ | ^NA^ |  |
| ^Evaluation of reported fertility preservation (…)^ | ^Patel^ | ^2020^ | ^JAMA^ | ^6976^ | ^Cross-sectional study^ | ^ASCO QOPI data set: documentation or FP referral^ | ^2015-2019^ | ^Men and women aged between 18 and 40 years (50 for men) All cancers Treatment with chemotherapy^ | ^44% (56% women)^ | ^NA^ | **^yes^** | ^NA^ | ^NA^ | ^NA^ | ^NA^ | **^yes^** | ^NA^ | **^yes^** | ^NA^ | | ^NA^ | ^NA^ | **^no^** |  |
| ^Fertility couseling before cancer treatment (…)^ | ^Young^ | ^2019^ | ^Cancer^ | ^747^ | ^Cross-sectional study prospective^ | ^Questionnaire^ | ^2015-2017^ | ^Men and women aged between 18 and 40 years  All cancers (21% breast)^ | ^19%^ | ^12%^ | **^yes^** | **^yes^** | ^NA^ | **^yes^** | ^NA^ | ^NA^ | ^NA^ | ^NA^ | **^yes^** | | ^NA^ | ^NA^ | ^NA^ |  |
| ^Trends of socioeconomic disparities in (…)^ | ^Goodman^ | ^2012^ | ^Human Reprod^ | ^199^ | ^Retrospective cohort study^ | ^EHR reviewed^ | ^2008-2010^ | ^Men and women aged between 18 and 42 years  All cancers^ | ^20.6%^ | ^5.5%^ | **^yes^** | **^yes^** | ^NA^ | **^no^** | ^NA^ | ^NA^ | ^NA^ | **^no^** | **^no^** | | ^NA^ | ^NA^ | ^NA^ |  |
| ^Referral for fertility preservation couselling (…)^ | ^Bastings^ | ^2014^ | ^Human Reprod^ | ^1169^ | ^Retrospective observational study^ | ^EHR reviewed and questionnaire^ | ^2001-2013^ | ^Women aged 0 to 39 years Invasive cancer^ | ^9.8%^ | ^NA^ | **^yes^** | ^NA^ | ^NA^ | ^NA^ | ^NA^ | **^yes^** | ^NA^ | ^NA^ | **^yes^** | | ^NA^ | ^NA^ | **^yes^** |  |
